# Chemical Profiling and Bioactivity of Body Wall Lipids from *Strongylocentrotus droebachiensis*

**DOI:** 10.3390/md15120365

**Published:** 2017-11-24

**Authors:** Alexander N. Shikov, Into Laakso, Olga N. Pozharitskaya, Tuulikki Seppänen-Laakso, Anna S. Krishtopina, Marina N. Makarova, Heikki Vuorela, Valery Makarov

**Affiliations:** 1 Saint-Petersburg Institute of Pharmacy, Leningrad Region, Vsevolozhsky District, Kuzmolovo P 245, 188663 Saint-Petersburg, Russia; olgapozhar@mail.ru (O.N.P.); annakrishtopina@list.ru (A.S.K.); mmn2410@yandex.ru (M.N.M.); makarov.vg@doclinika.ru (V.M.); 2 Division of Pharmaceutical Biosciences, Faculty of Pharmacy, University of Helsinki, P.O. Box 56 (Viikinkaari 5E), FI-00014 Helsinki, Finland; into.laakso@helsinki.fi (I.L.); heikki.vuorela@helsinki.fi (H.V.); 3 VTT Technical Research Centre of Finland Ltd., P.O. Box 1000 (Tietotie 2), FI-02044 VTT Espoo, Finland; tuulikki.seppanen-laakso@vtt.fi

**Keywords:** sea urchin, body wall lipids, inhibition of p38 MAPK, COX, GC-MS, UPLC-ELSD

## Abstract

The lipids from gonads and polyhydroxynaphthoquinone pigments from body walls of sea urchins are intensively studied. However, little is known about the body wall (BW) lipids. Ethanol extract (55 °C) contained about equal amounts of saturated (SaFA) and monounsaturated fatty acids (MUFA) representing 60% of total fatty acids, with myristic, palmitic and eicosenoic acids as major SaFAs and MUFAs, respectively. Non-methylene-interrupted dienes (13%) were composed of eicosadienoic and docosadienoic acids. Long-chain polyunsaturated fatty acids (LC-PUFA) included two main components, n6 arachidonic and n3 eicosapentaenoic acids, even with equal concentrations (15 μg/mg) and a balanced n6/n3 PUFA ratio (0.86). The UPLC-ELSD analysis showed that a great majority of the lipids (80%) in the ethanolic extract were phosphatidylcholine (60 μg/mg) and phosphatidylethanolamine (40 μg/mg), while the proportion of neutral lipids remained lower than 20%. In addition, alkoxyglycerol derivatives—chimyl, selachyl, and batyl alcohols—were quantified. We have assumed that the mechanism of action of body wall lipids in the present study is via the inhibition of MAPK p38, COX-1, and COX-2. Our findings open the prospective to utilize this lipid fraction as a source for the development of drugs with anti-inflammatory activity.

## 1. Introduction

Sea urchins have wide distribution and they play an important role in ecosystem of both shallow and deeper waters of the ocean. A number of species of sea urchin are intensively utilized in food, pharmaceutical, and cosmetic industries. In 2015, the total world production of edible sea urchin was 71,229 tons [1]. Green sea urchin, *Strongylocentrotus droebachiensis*, is an edible species of the phylum *Echinodermata*, which is a typical inhabitant of the polar region of Russia, including the Barents Sea. The gonads are delicacies in many parts of the world and considered as highly valued seafood. Several studies have indicated that the gonads of *S. droebachiensis* are rich in important bioactive compounds like polyunsaturated fatty acids (PUFA), phospholipids, tocopherols, sterols [2,3,4], carotenoids [2,5], and amino acids [6,7]. Extract of gonad tissue has also revealed effective anti-inflammatory and antidiabetic properties [4].

After removal of gonads, the residual shells and spines (body wall, BW) of sea urchins which account for more than 40% of total body weight are discarded as waste. Previous studies have also shown that sea urchin BW contain polyhydroxynaphthoquinones. These pigments have evoked renewed interest as a promising source for the development of drugs. A number of bioactivities have been found, for example antiallergic [8], antidiabetic [9], antihypertensive [10], anti-inflammatory [11], antioxidant [12,13,14], cardioprotective [15], and hypocholesterolemic [16] effects.

Total concentration of pigments in sea urchin body wall is quite low (1.2–1.6 mg/g) [17], however, a purification method has been recently reported in order to improve the yield of shell pigments [18]. The inner layer of the body wall, on the contrary, is covered with a biomembrane, consisting of lipids. However, their profile has not been described yet. Marine lipids are unique sources of essential fatty acids, phospholipids, sterols, and alkoxyglycerols, and they have a broad pharmacological activity.

The aim of this study was to analyze the ethanolic extract of lipids underlying the body wall (BW) of green sea urchin by using gas chromatography-mass spectrometry (GC-MS) and ultra-performance liquid chromatography (UPLC-ELSD). In addition, the anti-inflammatory potential of BW lipids was investigated in vitro.

## 2. Results

### 2.1. Fatty Acid Composition of Body Wall (BW) Lipids

**Figure 1:**
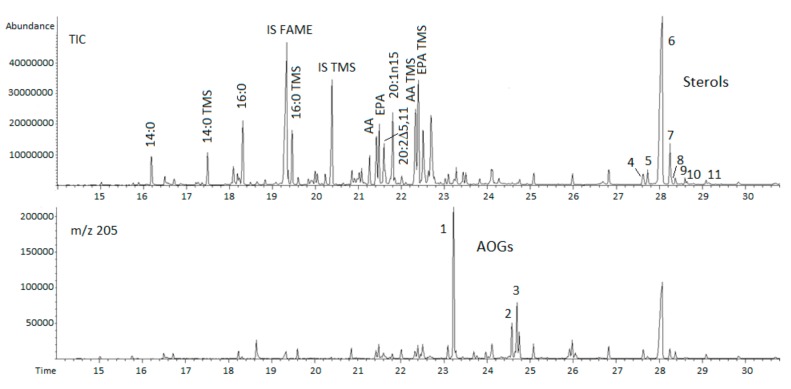
GC-MS analysis of transesterified and trimethylsilylated (TMS) fatty acids from ethanolic extract of BW lipids of sea urchin. Total ion (TIC) and extracted ion chromatogram (*m*/*z* 205) shows TMS derivatives of alkylglycerols (AOG; (1) 16:0-AOG, (2) 18:1-AOG, and (3) 18:0-AOG) and sterols (peaks 4–11, TIC): (4–5) unidentified sterols, (6) cholesterol, (7) desmosterol, (8) cholecalciferol as shoulder, (9) campesterol, (10) stigmasterol, and (11) clionasterol. Heptadecanoic acid (as FAME and TMS derivative) was used as internal standard (IS). Other peaks represent FAMEs and TMS ethers.

The ethanolic (95%) extract, which was prepared at 55 °C for 3 h, contained about equal amounts of saturated (SaFA) and monounsaturated fatty acids (MUFA) representing 60% of total fatty acids. Myristic, palmitic, and eicosenoic acids were the main SaFAs and MUFAs, respectively (Table 1 and Figure 1). The principal MUFA is unusual and this isomer is suggested to be 20:1n15. Among non-methylene-interrupted dienes (NMID, 13%), eicosadienoic (20:2) and docosadienoic (22:2) acids were characteristic. They are typical constituents of sea urchin among which 20:2Δ5,11 often appears as the most abundant isomer. In addition, n12 and n5 isomers of 18:1, 20:3n9 and cyclopropaneoctanoic and -decanoic acid 2-octyl methyl esters accounted 10 μg/mg.

The composition of long-chain polyunsaturated fatty acids (LC-PUFA) was characterized by two major components, n6 arachidonic (20:4n6; AA) and n3 eicosapentaenoic acids (20:5n3; EPA), with equal concentrations (15 μg/mg) and a balanced n6/n3 PUFA ratio (0.86). These LC-PUFAs possess important properties, since they act as the precursors of eicosanoids.

A very low amount of docosahexaenoic acid (22:6n3, DHA) was typical for BW lipid extract (Table 1), as well as the high proportion of free (22%) vs. esterified fatty acids. Quantitatively, free AA covered 1/3 of total AA and free EPA 1/4 of total EPA, respectively, reflecting decomposition of bound fatty acids during extraction procedure. The relatively high proportion of LC-PUFAs, n6 AA, and n3 EPA (20%), would suggest that the extract is rich in phospholipids.

### 2.2. Sterols and Alkoxyglycerols (AOG)

Sterol and AOG samples were analyzed by GC-MS as TMS-derivatives. BW lipid extract contained mainly cholesterol (50 μg/mg) (Figure 1 and Table 2). Minor non-cholesterol sterols included desmosterol, campesterol, stigmasterol, and clionasterol (gamma-sitosterol) which is a common constituent in oyster, for example [19]. For GC-MS analyses from abundant AOG sources, purified samples from unsaponifiable fraction have been used. Because of low concentration, extracted ion chromatogram (*m*/*z* 205) of silylated AOG was taken to confirm the peak location. This fragment is formed after cleavage between carbons 1 and 2 of the glycerol moiety [20]. Quantified AOG derivatives were chimyl (C16:0), selachyl (C18:1) and batyl alcohols (C18:0).

### 2.3. Lipid Classes

**Figure 2:**
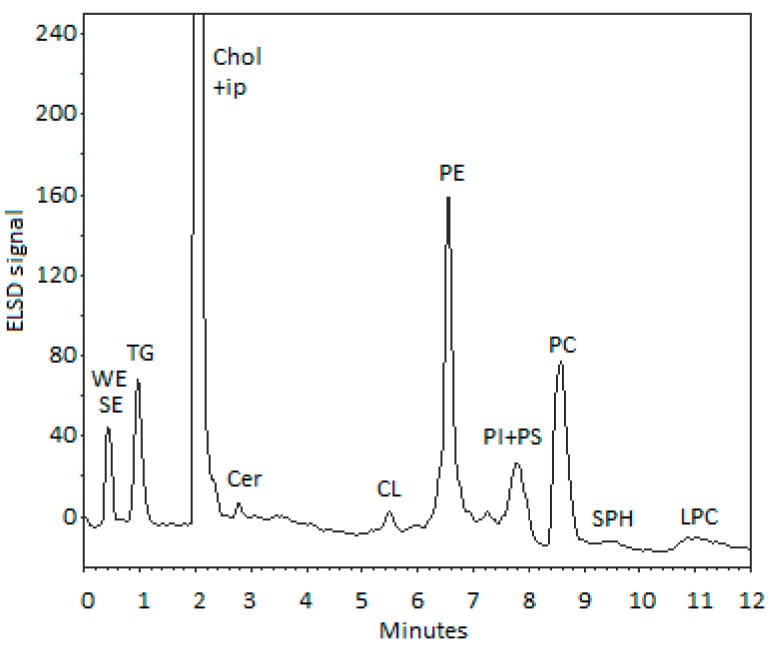
UPLC-ELSD chromatogram of BW lipids of ethanol (95%) extract of sea urchin. UPLC, ultra-performance liquid chromatography; ELSD, evaporative light scattering detector; WE, wax ester, SE, steryl ester; TG, triacylglycerol; Chol, cholesterol; ip, impurity; Cer, ceramide; CL, cardiolipin; PE, phosphatidylethanolamine; PI, phosphatidylinositol; PS, phosphatidylserine; PC, phosphatidylcholine; SPH, sphingomyelin; LPC, lysophosphatidylcholine.

The UPLC-ELSD analyses from the ethanol extract of BW lipids, shown in Figure 2 and Table 3, demonstrate high abundance of phospholipids (PL, 80%), especially those of phosphatidylcholine (PC, 60 μg/mg) and phosphatidylethanolamine (PE, 40 μg/mg). The proportion of neutral lipids remained less than 20%. It is clear that ethanol extracts polar PLs better than neutral lipids (NL) like triacylglycerols and cholesteryl esters. The fatty acid profile with relatively high content of PUFAs indicates that the fatty acids have mostly originated from PLs. Lysophosphatidylcholine (LPC) eluted late as a broad peak and covered about 8% of total lipids.

### 2.4. Bioactivity of Body Wall (BW) Lipids

Inflammation is a part of the body's normal response to infection and injury, extreme or inappropriate inflammation, on the contrary, is linked to the pathobiology of several diseases [21]. The anti-inflammatory effect of *S. droebachiensis* lipids of the extract was assessed in the human mononuclear U937 cells stimulated with lipopolysaccharide (LPS). The stimulation of U937 resulted in direct activation of MAPK p38. The results (Table 4) revealed that BW lipids dose-dependently inhibited MAPK p38. The most effective dose of the lipid extract was 0.033 μg/mL. In addition, BW lipids were clearly more potent than a specific MAPK p38 inhibitor SB203580 (1.88 μg/mL) at the doses of 0.0037–0.1 μg/mL. The COX-1 and COX-2 isoenzymes were inhibited by BW lipids dose-dependently with IC_50_ = 15.7 μg/mL and 21 μg/mL, respectively.

## 3. Discussion

The fatty acid composition of the ethanol extract of body wall (BW) lipids of sea urchin (Table 1 and Figure 1) was consistent with literature including non-methylene interrupted dienes (NMID) and unusual cyclopropane derivatives [2,22,23]. The principal PUFAs were n6 arachidonic (AA) and n3 eicosapentaenoic acids (EPA). The proportion docosahexanoic acid (22:6n3, DHA) in BW lipids, on the contrary, was very low as has been reported also by others [2,22]. In our previous study, gonads of sea urchin contained about 50 μg/mg of EPA and DHA [4]. Analysis of lipid classes showed two major phospholipids—i.e., phosphatidylcholine and phosphatidylethanolamine. This data is in general agreement with the phospholipids profile of gonads of *S. droebachiensis* [7].

Fats in human diet are responsible for severe health problems because of long-term intake of unbalanced proportions of SaFA, MUFA, n6, and n3 PUFA. The n6 and n3 PUFA intake favors too much n6 PUFA. This presupposes an adequate intake of PUFA precursors (n6 linoleic and n3 alpha-linolenic acids) to form long-chain LC-PUFAs (n6-AA and n3-EPA), which, in turn, act as eicosanoid precursors which determine the balance and effects of eicosanoids in the body [24]. Arachidonic acid is converted to thromboxane-type eicosanoids via cyclo-oxygenase enzyme, while EPA is converted to prostacycline, antagonizing the conversion of AA to eicosanoids. This would enhance anti-aggregatory and anti-inflammatory conditions [25].

From a biological point of view, the biomembrane covering the inner layer of the body wall plays an important protective role for survival of the sea urchin. Damage of the body wall will follow with inflammation. Mitogen-activated protein kinases (MAPKs) are among the most important molecules in the signaling pathways among which MAPK p38 signaling plays an essential role in regulating cellular processes, especially inflammation [26]. In our study, we observed 88% of MAPK p38 inhibition by body wall (BW) lipids at a very low dose of 0.033 μg/mL (Table 4). The inhibition of MAPK p38 might be attributed to the different active compounds of BW lipids. Ait-Said et al. [27] established that EPA, unlike DHA, failed to inhibit nuclear factor-κB (NF-κB) activation, and suppressed MAPK p38 phosphorylation in IL-1β stimulated human pulmonary microvascular endothelial cells. The anti-inflammatory properties of EPA in LPS-stimulated BV2 microglia cells were mediated by downregulation of NF-κB and MAPKs such as ERK, p38, JNK, and Akt activation [28].

Cyclooxygenase-2 (COX-2) isoenzyme could be induced by a wide range of proinflammatory agents. Prostaglandin-dependent amplification of COX-2 is hypothesized to be an important part of sustained proliferative and chronic inflammatory conditions [29]. EPA as well as DHA effectively inhibited COX-2 expression in LPS-stimulated HUVEC endothelial cells [30]. Recently, we have reported that lipid rich fraction from gonads of *S. droebachiensis* inhibited COX-2 with IC_50_ = 49 μg/mL, but was not effective against COX-1 isoform [4]. It is important to note that, in our current study, body wall lipids were more effective and inhibited COX-2 in lower dose and inhibited COX-1 with IC_50_ = 15.7 μg/mL.

Low amounts of ether-bonded alkoxyglycerols were also found in ethanolic extract of BW lipids (Figure 2 and Table 3). The total amount of AOG was less than 0.2% which would correspond about the level in human milk and plasma lipids, for example [31]. By using the present UPLC-ELSD method, however, it was not possible to detect glycerophospholipid-based alkyl- and alkenylacyl lipids, since their analysis first requires the separation of phospholipid subclasses. These lipids, such as glycerophosphatidylethanolamine and -choline, are known to have important activities.

Biological activity of alkoxyglycerols has been known already more than half a century. Some of the activities, such as anti-inflammatory effects and protection against radiation damage, are still under study together with the more recent interest in the possible cell-signaling properties of phospholipid-based ether-bonded compounds. These lipids have shown multiple pharmacological activities such as anti-cancer [32], wound healing [33], and immunostimulatory effects [34]. It has been demonstrated that AOG differentially modulate LPS-mediated MAPK and NF-κB signaling in adipocytes and that they do not activate signaling in the absence of LPS. Saturated alkyl chain increased LPS-mediated activation of the MAPK signaling, which could cause the expression of inflammatory genes. Conversely, unsaturated alkyl chain decreased LPS-mediated activation of the MAPK and NF-κB signaling [35].

Taking into account all these aspects, we can hypothesize that the lipids containing n3 PUFA, especially EPA, could contribute anti-inflammatory activity. Besides the content of neutral lipid alkoxyglycerols, it is necessary to confirm the occurrence of alkyl- and alkenylacyl lipids and fatty acid compositions of individual phospholipid classes in BW lipid extracts.

## 4. Material and Methods

### 4.1. Sample Preparation and Extraction of Lipids

Green sea urchins, *Strongylocentrotus droebachiensis*, were harvested in the Barents Sea in 2016 by divers. After removal of gonads, coelomic fluid, and internal organs, the shells with spines were washed in cold tap water, dried at 4 °C for two days and stored in a dark place. The shells with spines (20 g) were ground and macerated with 160 mL of 95% ethanol for 3 h with constant stirring at 55 °C. The extract was filtered and evaporated into dryness by rotary evaporator (IKA RV 10; IKA^®^-Werke GmbH & Co. KG, Staufen, Germany). The yield of the body wall lipids was 1.2%.

### 4.2. Chemical Analyses

#### 4.2.1. Analysis of Fatty Acids, Sterols and Alkoxyglycerols by GC-MS

Analyses of bound and free (FFA) fatty acids were carried out by using the method described previously [4]. Shortly, the lipid extract, spiked with internal standards (IS) TG(17:0/17:0/17:0) and FFA 17:0, were transesterified with 0.5 N sodium methoxide at 45 °C for 5 min. After acidification, fatty acid methyl esters (FAMEs) as well as FFAs were extracted with petroleum ether. The method enabled the esterification of bound fatty acids from neutral lipids and also from phospholipids [36]. The mixtures of FAMEs and FFAs were used as reference substances.

The analyses were performed on an Agilent 7890A GC mounted with Gerstel MPS injection system and an Agilent 5975C mass selective detector. The column was an Agilent FFAP silica capillary column (25 m × 0.2 mm × 0.3 μm) and helium was used as the carrier gas. The oven temperature raised from 70 °C to 235 °C, with a total run time of 30 min. The temperatures of the injector and MS source were 220 and 230 °C, respectively, and the data were collected in EI mode (70 eV) at a mass range of *m*/*z* 40–600.

After analyzing the composition of FAMEs by GC-MS, the same samples were derivatized to determine the contents of FFAs, cholesterol, and minor sterols. Analyses of alkoxyglycerols (AOG) were done according to a previous method [31]. Samples were evaporated, re-dissolved in dichloromethane, and silylated with MSTFA [*N*-Methyl-*N*-(trimethylsilyl)-trifluoroacetamide] (Pierce, Rockford, IL, USA) at 80 °C for 20 min. TMS-derivatives were analyzed on an Rtx-5-ms column (15 m × 0.25 mm × 0.25 μm) (Restek, Bellefonte, PA, USA). The split ratio was 20:1 and the oven temperature was programmed to go from 70 (1 min) to 270 °C at a rate of 10 °C/min, the total run time was 30 min. The data was collected by MSD ChemStation software (Agilent Technologies, Inc., Santa Clara, CA, USA). Identification of the compounds was based on retention times of reference substances, library comparisons (The Wiley^®^ Registry of Mass Spectral Data, John Wiley and Sons, Inc., New York, NY, USA; NIST 08 spectral library, National Institute of Standards and Technology, Gaithersburg, MD, USA) and on literature data.

#### 4.2.2. Analysis of Lipid Classes by UPLC-ELSD

The same lipid extract as above (without derivatization) was analyzed on a Waters Acquity^TM^ H-class UPLC (ultra-performance liquid chromatograph) equipped with an evaporative light scattering detector (ELSD) by modifying previous conditions [37]. Separation of the lipid classes was carried out on a Waters Spherisorb silica column (3 μm, 100 × 2.1 mm I.D.). The gradient solvent system consisted of (A) iso-octane-tetrahydrofurane (99:1), (B) 2-propanol-dichloromethane (3:2) and (C) 2-propanol-water (1:1) with an analysis time of 20 min. The temperature of the drift tube was 40 °C and air flow 50 psi. The multigradient system started from 100% A, the proportion of A decreased to 32%, that of B increased to 52% and simultaneously that of C (containing water) increased to 16%. Stable retention times were obtained by keeping continuous cycle running. The flow rate was 0.800 mL/min and the injection volume 2 μL. The temperature of the sample manager was 10 °C.

### 4.3. Biological Assays

#### 4.3.1. Cell Lines and Cell Culture

The human mononuclear U937 cells were purchased from the Russian Collection of Cell Culture (Institute of Cytology of Russian Academy of Science, Saint-Petersburg, Russia), and maintained at 37 °C in a humidified 95% air and 5% CO_2_ in RPMI1640 supplemented with 2 mM glutamine, 10% heat-inactivated FBS, 100 U/mL penicillin, and 100 μg/mL streptomycin. BW lipids were dissolved in dimethyl sulfoxide (DMSO) as a stock solution at a 10 mg/mL concentration, and the stock solution was then diluted with the medium to the desired concentration prior to use. Cells derived from the freeze-down batch were thawed, grown, and seeded (106 cells per well) onto 12-well tissue culture plates and cultured in medium for 24 h. The cells were then stimulated with 1 μg/mL *Escherichia coli* LPS (Sigma-Aldrich, St. Louis, MO, USA) at 37 °C for 1 h. After that the cells were treated with SB203580 (Sigma-Aldrich, St. Louis, MO, USA) and various concentrations of BWL at 37 °C for 1 h.

#### 4.3.2. Western Blotting

Cells were washed in cold (4 °C) phosphate-buffered saline (PBS; 0.5 mol/L sodium phosphate, pH 7.5) and separated by centrifugation (Hermle Labortechnik, Germany) at 1500 rpm^−1^ for 5 min at 4 °C, harvested by gentle scraping, and used to prepare total protein or nuclear extracts. Cells were treated with lysis buffer—1 mol/L Tris-HCl pH 7.5, 1.5 mol/L NaCl, 10% Triton X-100, 0.2 mol/L Na_3_VO_4_, 1 mol/L NaF, 0.2 mol/L EDTA, phenylmethylsulphonyl fluoride (PMSF), Abcam’s protease inhibitor cocktail, and Abcam’s phosphatase inhibitor cocktail—for 20 min at 4 °C. The lysates were then clarified by centrifugation at 15,000 rpm^−1^ for 15 min at 4 °C and the supernatant was collected.

The protein concentrations of the extracts were determined using the [38] with an XMark spectrophotometer (Bio-Rad, Hercules, CA, USA). For Western blot analysis, 40 μg of protein were desaturated by boiling with Laemmli buffer (5 min at 100 °C) and subjected to 4–14% SDS-polyacrylamide gels, and transferred to nitrocellulose membrane membranes (Bio-Rad) by electroblotting. The membranes were blocked with 5% non-fat dry milk in PBS with Tween 20 buffer (PBS-T) (Tris-HCl (pH 7.5), 1.5 mol NaCl, and 0.1% Tween 20) for 1 h at room temperature. Membranes were then incubated overnight at 4 °C with the primary antibodies, probed with enzyme-linked secondary antibodies, and visualized using a chemiluminescent detection with LumiGLO^®^ reagent (Cell Signaling Technology, Danvers, MA, USA) according to the manufacturer’s instructions. After detection, the membranes were scanned (Epson Perfection V330 Photo, Seiko Epson Corporation, Nagano, Japan) and processed with Scion Image software (Alpha 4.0.3.2, Scion, Fredrick, MD, USA). The band intensities were used for calculations. Phospho-p38 MAPK antibody, rabbit, p38 MAPK XP rabbit mAb, β-actin rabbit mAb, and anti-rabbit IgG, HRP-linked antibody were from Cell Signaling Technology (Danvers, MA, USA).

#### 4.3.3. Assessment of Cyclooxygenase Activity

Inhibition of human recombinant cyclooxygenase COX-1 and COX-2 (Cayman Chemical, Ann Arbor, MI, USA) was assessed according to the manufacturer’s instructions. Indomethacin (1 μg/mL) from Sigma (St Louis, MO, USA) was used as reference. The BWL was dissolved in DMSO prior to analysis.

### 4.4. Statistical Analysis

Data were analyzed using Statistica version 10.0. All biological assay data are presented as mean ± SEM or mean ± SD. Differences among groups were evaluated by one way analysis of variance (ANOVA) and post-hoc Tukey’s test. In all comparisons, *p* < 0.05 was accepted as statistically significant.

## 5. Conclusions

To the best of our knowledge, this is the first time when the profile of body wall lipids is reported. It can be assumed that the mechanism of action of body wall lipids in the present study is via the inhibition of MAPK p38, COX-1, and COX-2. Our findings open the potential to utilize this lipid fraction as a source for the development of drugs with anti-inflammatory activity. Further- more, the anti-inflammatory properties of the lipid extract may be useful for ameliorating neuro- degenerative diseases, as well as suppressing LPS-induced shock.

## Figures and Tables

**Table 1:** The concentrations (μg/mg; mean ± SD, *n* = 3) and relative amounts (%) of bound and free fatty acids in ethanolic extract of BW lipids of sea urchin determined by GC-MS.

Fatty Acids	Bound Fatty Acids as FAME	%	FFA	%
C10:0–C13:0	0.4 ± 0.1	0.3	-	-
C14:0	15.1 ± 0.7	9.6	3.7 ± 0.1	8.4
C15:0	1.5 ± 0.1	0.9	0.5 ± 0.1	1.1
C16:0	21.9 ± 1.2	13.9	5.0 ± 0.2	11.4
C18:0	4.0 ± 0.2	2.5	2.1 ± 0.1	4.8
C19:0	1.0 ± 0.1	0.6	-	-
C20:0	0.6 ± 0.1	0.4	-	-
Σ SaFAΣ	44.5 ± 2.1	28.2	11.3 ± 0.3	25.7
C14:1n5	1.1 ± 0.1	0.7	-	-
C16:1n9	0.9 ± 0.1	0.6	-	-
C16:1n7	7.8 ± 0.6	4.9	1.9 ± 0.1	4.3
C16:1n5	4.5 ± 0.2	2.8	-	-
C18:1n9	3.0 ± 0.2	1.9	1.4 ± 0.1	3.2
C18:1n7	4.2 ± 0.2	2.7	2.9 ± 0.1	6.6
C20:1n15	16.7 ± 0.8	10.6	-	-
C20:1n9	5.2 ± 0.3	3.3	8.2 ± 0.4	18.6
C20:1n7	1.3 ± 0.1	0.8	-	-
C22:1n9	4.3 ± 0.2	2.7	-	-
Σ MUFA	49.0 ± 1.3	31.0	14.4 ± 0.4	32.7
20:2Δ5,11	12.2 ±1.2	7.7	-	-
20:2Δ5,13	3.0 ± 0.5	1.9	-	-
22:2Δ7,13	0.9 ± 0.2	0.6	-	-
22:2Δ7,15	4.7 ± 0.4	3.0	-	-
Σ NMID	20.9 ± 1.9	13.2	-	-
C18:2n6	1.5 ± 0.1	0.9	5.2 ± 0.2	11.8
C20:2n6	1.9 ± 0.1	1.2	-	-
C20:3n6	0.9 ± 0.1	0.6	-	-
C20:4n6	15.8 ± 0.6	10.0	7.3 ± 0.1	16.6
Σ n6 PUFA	20.1 ± 1.0	12.7	12.5 ± 0.8	28.4
C18:3n3	1.2 ± 0.1	0.8	-	-
C18:4n3	2.3 ± 0.1	1.5	-	-
C20:3n3	2.3 ± 0.2	1.5	-	-
C20:4n3	0.6 ± 0.1	0.4	-	-
C20:5n3	15.2 ± 0.7	9.6	5.8 ± 1.0	13.2
C22:5n3	0.2 ± 0.1	0.1	-	-
C22:6n3	1.7 ± 0.1	1.1	-	-
Σ n3 PUFA	23.5 ± 1.1	14.9	5.8 ± 1.0	13.2
Σ Fatty acids	158.0 ± 5.3	100.0	44.0 ± 1.8	100.0
n6/n3 PUFA	0.86		2.16	

FAME, fatty acid methyl ester; FFA, free fatty acid; SaFA, saturated fatty acid; MUFA, mono-unsaturated fatty acid; NMID, non-methylene-interrupted diene; PUFA, polyunsaturated fatty acid.

**Table 2:** Sterol and alkoxyglycerol (AOG) content (μg/mg; mean ± SD, *n* = 3) of ethanolic extract of BW lipids of sea urchin. Sterols and AOGs were determined as TMS ethers by GC-MS.

Sterols and AOGs	μg/mg
Cholesterol	50.3 ± 2.8
Non-cholesterol sterols *	10.6 ± 0.5
C16:0-AOG	1.0 ± 0.1
C18:1-AOG	0.4 ± 0.1
C18:0-AOG	0.3 ± 0.1
Σ Alkoxyglycerols	1.7 ± 0.2

* Non-cholesterol sterols include desmosterol, campesterol, stigmasterol, clionasterol, and two unidentified sterols (Figure 1).

**Table 3:** Concentration of major lipid classes (μg/mg, mean ± SD; *n* = 3) and their relative amounts (%) in ethanolic extract of BW lipids of sea urchin. Analyses were carried out by UPLC-ELSD.

Lipid Classes	μg/mg	%
WE + SE	14.6 ± 0.7	8.4
TG	15.9 ± 1.0	9.1
Σ Neutral lipids	30.5 ± 1.6	17.5
PE	38.6 ± 1.1	22.2
PI + PS	27.8 ± 0.1	16.0
PC	63.3 ± 1.7	36.4
LPC	13.5 ± 0.5	7.8
Σ Phospholipids	143.2 ± 0.7	82.5
Σ Total lipids	173.7 ± 2.3	100.0

WE, wax ester; SE, steryl ester; TG, triacylglycerol; PE, phosphatidylethanolamine; PS, phosphatidylserine; PI, phosphatidylinositol; PC, phosphatidylcholine; LPC, lysophosphatidylcholine.

**Table 4:** Effect of body wall (BW) lipids on the phosphorylation of MAPK p38 in the human mononuclear U937 cells Mean ± SEM, (*n* = 6).

Sample, Concentration	Percentage of MAPK p38 (%)
Intact cells (no stimulation with LPS)	23.0 ± 1.2
Control cells stimulated with LPS (1 μg/mL)	100
SB203580 (1.88 μg/mL) + LPS	30.0 ± 1.7
BWL (10 μg/mL) + LPS	59.0 ± 1.3
BWL (5 μg/mL) + LPS	53.0 ± 1.9
BWL (1 μg/mL) + LPS	49.0 ± 0.9
BWL (0.5 μg/mL) + LPS	38.0 ± 1.6
BWL (0.1 μg/mL) + LPS	17.0 ± 1.5
BWL (0.033 μg/mL) + LPS	12.0 ± 0.5
BWL (0.011 μg/mL) + LPS	21.0 ± 1.7
BWL (0.0037μg/mL) + LPS	27.0 ± 0.7
BWL (0.0012 μg/mL) + LPS	38.0 ± 1.6
BWL (0.0004 μg/mL) + LPS	52.0 ± 1.2
